# *Protanthomyza grimaldii* sp. nov., a further member of the extinct subfamily Protanthomyzinae (Diptera, Anthomyzidae) from Baltic amber

**DOI:** 10.3897/zookeys.973.51435

**Published:** 2020-10-05

**Authors:** Jindřich Roháček

**Affiliations:** 1 Silesian Museum, Nádražní okruh 31, CZ-746 01 Opava, Czech Republic Silesian Museum Opava Czech Republic

**Keywords:** Anthomyzid flies, adult morphology, Eocene, new extinct species, relationships, taxonomy, Tertiary

## Abstract

A new fossil species, *Protanthomyza grimaldii***sp. nov.** (Diptera, Anthomyzidae), is described from Baltic amber (Eocene, 48–34 Ma) based on two (male and female) inclusions. It is the ninth species of the †genus *Protanthomyza* Hennig, 1965 and †subfamily Protanthomyzinae Roháček, 1998. Adult morphology of *P. grimaldii***sp. nov.** revealed that the rich chaetotaxy of the thoracic pleuron, two anal veins and presence of the anteroventral process of the epandrium are plausibly shared by all species of *Protanthomyza*. Relationships of the new species, which belongs to a group lacking the ctenidial spine on the fore femur, are discussed.

## Introduction

Fossil taxa of Anthomyzidae (Diptera) were reviewed by Roháček (2013a), with one species subsequently added (Roháček 2014). A total of 12 ancient (all Tertiary) valid and named species belonging to four genera are currently recognized. The majority of fossil species of Anthomyzidae were described from Baltic amber (48–34 Ma) and only one, viz. *Grimalantha vulnerata* Roháček, 1998, originates from Dominican amber (Miocene, 18–16 Ma). Only four of the ancient species belong to the “modern” subfamily Anthomyzinae Czerny, 1903, viz. the above *Grimalantha vulnerata* from the Miocene (see Roháček, 1998) and *Lacrimyza lacrimosa* Roháček, 2013, *L. christelae* Roháček, 2013 and *Reliquantha eocena* Roháček, 2014 from the Eocene. The latter species belongs to a genus originally described by Roháček (2013b) for an extant species, *R. variipes* Roháček, 2013 from Great Britain. All remaining ancient Anthomyzidae are members of the exclusively fossil subfamily Protanthomyzinae Roháček, 1998 and belong to its only genus *Protanthomyza* Hennig, 1965. Eight named species of this genus are currently recognized, viz. *P. collarti* Hennig, 1965, *P. hennigi* Roháček, 2013, *P. hoffeinsorum* Roháček, 2013, *P. krylovi* Roháček, 2013, *P. loewi* Roháček, 2013, *P. meunieri* Roháček, 2013, *P. presli* Roháček, 2013 and *P. tschirnhausi* Roháček, 2013, all of which were treated in detail by Roháček (2013a). However, Roháček (2013a: 451) also presented the diagnosis of one more new species of *Protanthomyza* which was left unnamed due to insufficient material (a single female with some parts of the body obscured). This unnamed species was considered distinctly different from all other known members of the genus and, therefore, it was also counted among them for the estimation of species diversity of Anthomyzidae in the Eocene Baltic amber forest ecosystem (Roháček 2013a: 470). Recently, Mrs. Christel Hoffeins purchased a nicely preserved *Protanthomyza* inclusion and provided it for study. Detailed examination of this specimen revealed it to be the formerly unknown male conspecific with the unnamed “*Protanthomyza* sp. nov.” female dealt with in Roháček (2013a). To supplement the latter monographic treatment, this new species is described in detail below, including the re-examination of the above female specimen. This is only the third fossil species of *Protanthomyza* (and the fourth of all ancient Anthomyzidae) in which both sexes are known.

## Material and methods

### Material

Two amber pieces with 2 anthomyzid inclusions were examined. Abbreviations of amber sources and depositories: **AMNH** – American Museum of Natural History, New York, USA; **CCHH** – collection of C. and H. W. Hoffeins, Hamburg, Germany; **CMTB** – collection of M. von Tschirnhaus, Bielefeld, Germany; **SDEI** – Senckenberg Deutsches Entomologisches Institut (Senckenberg German Entomological Institute) in Müncheberg, Germany.

### Preparation of amber specimens

The methods of preparation of amber stones with fly inclusions were described in detail by von Tschirnhaus and Hoffeins (2009). The amber specimens examined had already been cut out of the original stones, ground and polished as close and as parallel as possible to the frontal, dorsal and lateral sides of the fly and one of them had been subsequently embedded in artificial resin (also ground and polished) (Hoffeins 2001) to facilitate its stereoscopic investigation. This process left a significant cut portion of one stone bearing syninclusions, which remains deposited in CCHH.

### Techniques of investigation

The amber inclusions were examined, drawn and measured using two types of binocular stereoscopic microscopes (Reichert, Olympus). Legs were drawn on squared paper using a Reichert binocular microscope with an ocular screen. The specimens were either photographed by a Canon EOS 60D digital camera with Canon MP-E 65 mm 1–5× macro lens or by a Canon EOS 5D Mark III digital camera with a Nikon CFI Plan 10×/0.25NA 10.5mm WD objective attached to a Canon EF 70–200 mm f/4L USM zoom lens. The specimen photographed by means of the latter equipment was repositioned upwards between each exposure using a Cognisys StackShot Macro Rail and the final photograph was compiled from multiple layers (35) using Helicon Focus Pro 7.0.2. The final images were edited in Adobe Photoshop CS6. Some illustrations were drawn from these and some other macrophotographs and details were inked based on direct observation at higher magnification using a binocular microscope. Measurements: Six characteristics were measured – body length (measured from anterior margin of head to end of cercus, thus excluding the antenna), wing length (from wing base to wing tip), wing width (maximum width), index *Cs_3_* : *Cs_4_* (= ratio of length of 3^rd^ costal sector : length of 4^th^ costal sector), index *r-m\dm-cu* : *dm-cu* (= ratio of length of section between *r-m* and *dm-cu* on cell *dm* : length of *dm-cu*) and index *r-m\dm-cu* : *CuA_1_* (= ratio of length of section between *r-m* and *dm-cu* on cell *dm*: length of apical portion of *CuA_1_*).

**Morphological terminology** follows that used in Roháček (2006, 2009) and Roháček and Barber (2016), including terms of the male hypopygium to be in continuation with Roháček (2013a), except where “orbit” is replaced with “orbital plate”. Male terminalia terminology is largely based on the “hinge” hypothesis of the origin of the eremoneuran hypopygium, re-discovered and documented by Zatwarnicki (1996) and, therefore, the following alterations of terms of the male genitalia (against those used by other hypotheses) need to be listed (terms used here first): epandrium = periandrium, gonostylus = surstylus. Morphological terms of the male abdomen and terminalia are depicted in Figs 8, 9, those of the female abdomen in Fig. 13. The synonymous morphological terms of adult structures and their abbreviations as used in the recent manual of Afrotropical Diptera (Cumming and Wood 2017) are given in parentheses in the list of abbreviations below.

### Abbreviations of morphological terms used in text and/or figures

***A_1_*** first anal vein (= anterior + posterior branch of cubital vein, *CuA+ CuP*)

***A_2_*** second anal vein (= first branch of anal vein, *A_1_*)

***ac*** acrostichal (seta) (*acr*)

***ar*** arista

***avp*** anteroventral process of epandrium;

***C*** costa

***ce*** cercus

***CuA_1_*** cubitus (= fourth branch of media, *M_4_*)

***dc*** dorsocentral (seta)

***dm*** discal medial cell

***dm-cu*** discal medial-cubital (= discal medial, *dm-m*) cross-vein

***ep*** epandrium

***f_1_***, ***f_2_***, ***f_3_*** fore, mid, hind femur

***ha*** haltere

***hu*** humeral (= postpronotal, *pprn*) (seta)

***lbl*** labellum

***M*** media (= first branch of media, *M_1_*)

***mspl*** mesopleural (= anepisternal, *anepst*) (seta)

***npl*** notopleural (seta)

***oc*** ocellar (seta)

***ors*** orbital (seta) (*orb*)

***pa*** postalar (seta) (*pal*)

***pk*** preapical kink

***plp*** maxillary palpus

***poc*** postocular (setulae)

***ppl*** propleural (= proepisternal + proepimeral, *prepst* + *prepm*) (seta)

***prs*** presutural (= presutural intraalar, *ial*) (seta)

***prsc*** prescutellar acrostichal (seta)

***pvt*** postvertical (= postocellar, *poc*) (seta)

***R_1_***, ***R_2__+3_***, ***R_4+5_*** 1^st^, 2^nd^, 3^rd^ branches of radius

***r-m*** radial-medial cross-vein

***S1–S8*** abdominal sterna

***sa*** supraalar (seta) (*spal*)

***sc*** scutellar (seta) (*sctl*)

***Sc*** subcosta

***stpl*** sternopleural (= katepisternal, *kepst*) (seta)

***T1–T8*** abdominal terga

***t_1_***, ***t_2_***, ***t_3_*** fore, mid, hind tibia

***vi*** vibrissa (*vb*)

***vte*** outer vertical (seta) (*o vt*)

***vti*** inner vertical (seta) (*i vt*)

## Systematic palaeontology

### Class Insecta Linnaeus, 1758

**Order Diptera Linnaeus, 1758**

**Superfamily Opomyzoidea Fallén, 1820**

**Family Anthomyzidae Czerny, 1903**

**Subfamily Protanthomyzinae Roháček, 1998**

#### Genus Protanthomyza

Taxon classificationAnimaliaDipteraAnthomyzidae

Hennig, 1965

2E50143B-0BCB-5B3D-AA7A-9B23ACCACF1B

##### Type species.

*Protanthomyza collarti* Hennig, 1965; Baltic amber (Eocene).

#### Protanthomyza grimaldii sp. nov.

Taxon classificationAnimaliaDipteraAnthomyzidae

E5AF0B3A-36C3-549A-AAC7-9C1FE665BEE0

http://zoobank.org/D95A90D3-4F55-46E2-814A-4D8117555C47

Figures 1–2 , 3 , 4 , 5–9 , 10, 11 , 12–14

Protanthomyza sp. nov.: Roháček 2013a: 451–452, fig. 7E, F (diagnosis, female only).

##### Etymology.

The species is dedicated to Prof. Dr. David Grimaldi (New York, U. S. A.), the distinguished American dipterist and palaeontologist, in recognition of his valuable contribution to the knowledge of amber fossil flies, including acalyptrates (largely from Dominican amber).

##### Type material.

***Holotype*** ♂ labelled “Faszination Bernstein, Christel Hoffeins, Hans Werner Hoffeins” (framed on obverse), ”1040-5a Diptera: Acalyptratae, Anthomyzidae ♂” (handwritten by C. Hoffeins, on reverse), “Baltic amber, Russia: Kaliningrad region, Yantarny”, “obtained in May 2010 from Dr. Andrey Krylov, Kaliningrad, Russia”, and “Holotypus ♂, *Protanthomyza grimaldii* sp.n., J. Roháček det. 2020” (red label) [amber piece embedded in polyester resin, size 8.6 × 7.9 × 4.8 mm], deposited in SDEI (inventory number Dip-00821). The original amber stone (in form of an icicle = Zapfenschlaube in German), size about 45 × 20 × 11 mm, with multiple layers and aggregation of inclusions, was cut in two pieces; that with the inclusion of *P. grimaldii* (No. 1040-5a) was separated, manually prepared and embedded in polyester resin by H. W. Hoffeins in August 2015. Syninclusions in 1040-5a: 1 stellate hair, pollen grains. Syninclusions in the remaining part (1040-5b, deposited in CCHH): Diptera: Empididae: 1 *Rhamphomyia* sp. female; 2 Mycetophilidae males + fragment; 1 Simuliidae; 2 Chironomidae female and male; Trichoptera, not identifiable; Coleoptera fragment; Araneae fragment; stellate hairs. Paratype ♀ labelled “47b-1, (Baltic A.), 15.1 × 10.7 × 3.3 [mm]” (handwritten), “*Protanthomyza* sp.n. ♀, J. Roháček det. 2011” (yellow label) and “Paratypus ♀, *Protanthomyza grimaldii* sp.n., J. Roháček det. 2020” (yellow label) [shape of stone irregularly pentagonal, thin], temporarily held in CMTB; it will be deposited in AMNH. Syninclusions: only stellate hairs.

##### Type locality and age.

Russia: Kaliningrad region, Yantarny mine. Middle to Late Eocene, 48–34 Ma (cf. Seyfullah et al. 2018).

##### Diagnosis.

Ocellar triangle delimited by a groove; frontal triangle not delimited; 1^st^ antennal flagellomere normal, not enlarged; arista bare; 3 or 4 *dc* macrosetae; *f_1_* without a ctenidial spine; male epandrium elongate and posteriorly tapered, with a robust, hook-like, curved anteroventral process; female T7 short; female S6 and S7 broadly transverse.

##### Description.

**Figures 1, 2.**
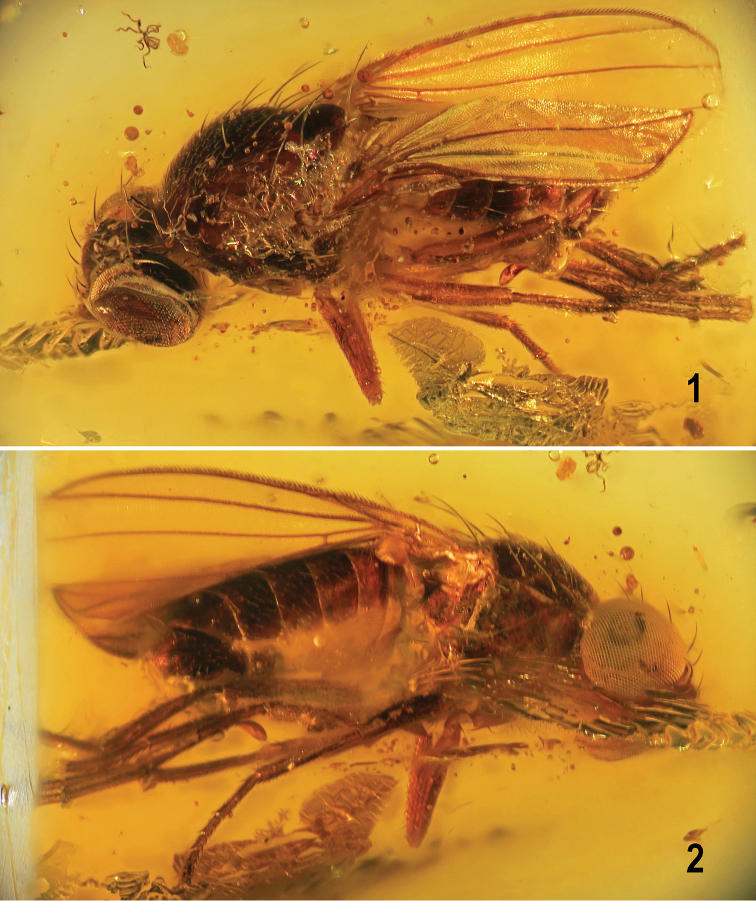
*Protanthomyza grimaldii* sp. nov., holotype male (Baltic amber) **1** entire specimen, left laterodorsal view **2** ditto, right lateroventral view. Body length ca 2.6 mm. Photographs by J. Roháček.

**Male** (Figs 1, 2). Total body length ca 2.6 mm; general colour brown to blackish brown; only head and some extremities partly ochreous to yellow.

**Figure 3.**
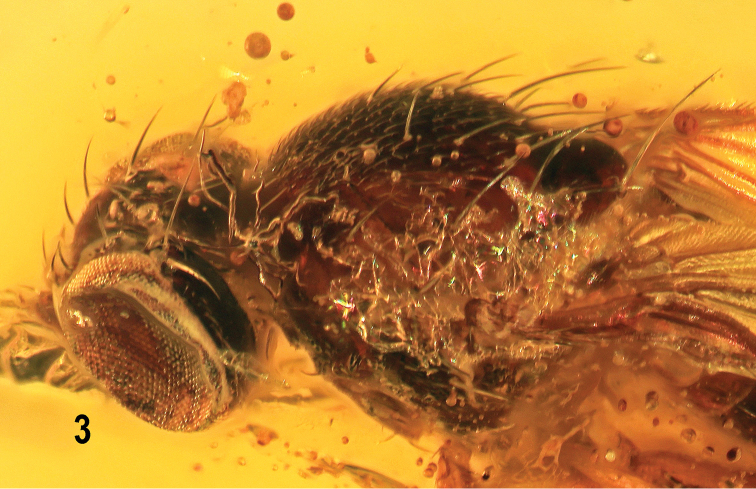
*Protanthomyza grimaldii* sp. nov., holotype male (Baltic amber). Head and thorax, left laterodorsal view. Photograph by J. Roháček.

**Figure 4.**
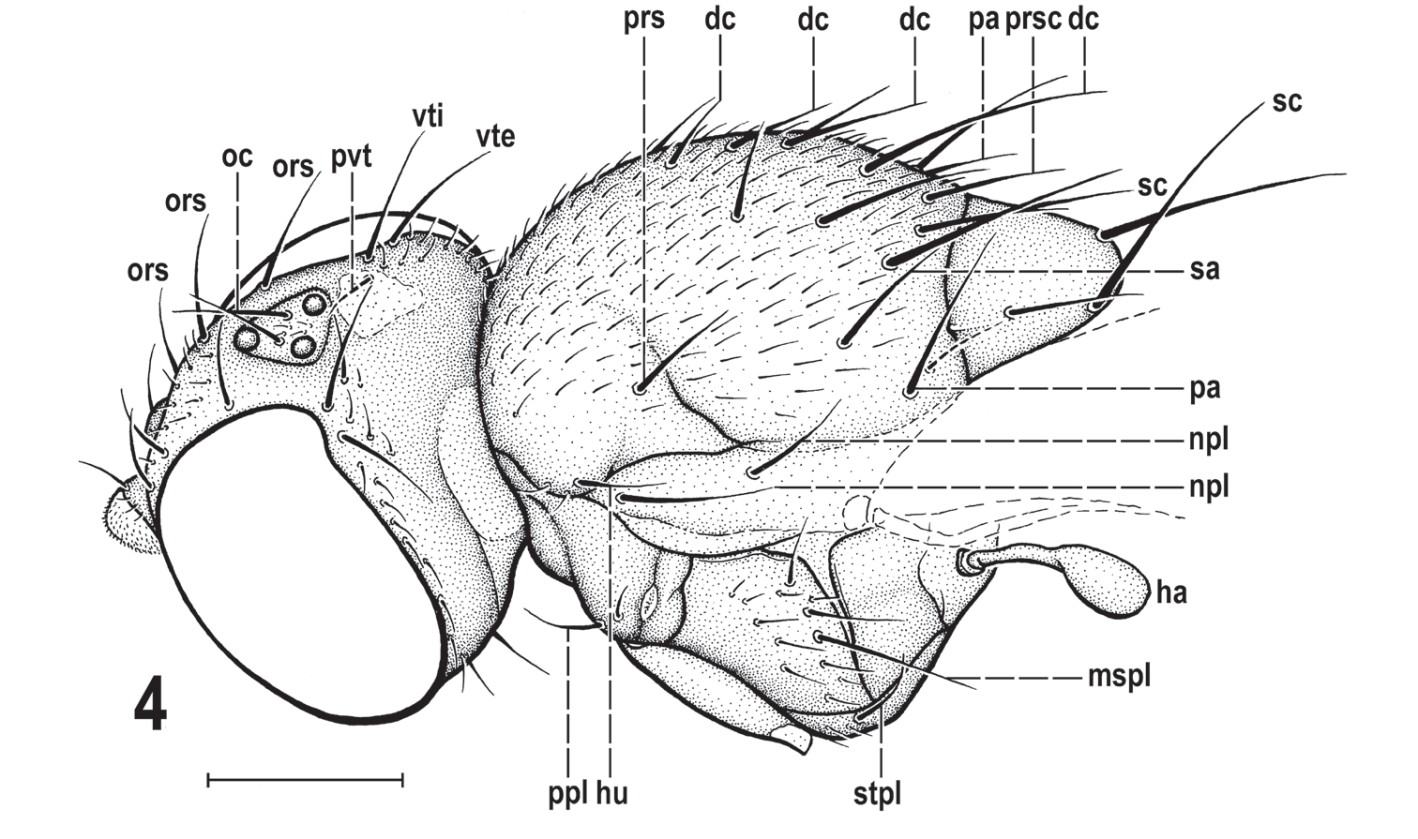
*Protanthomyza grimaldii* sp. nov., holotype male (Baltic amber). Head and thorax, left laterodorsal view. Scale bar: 0.3 mm. For abbreviations see p. 4.

**Figures 5–9.**
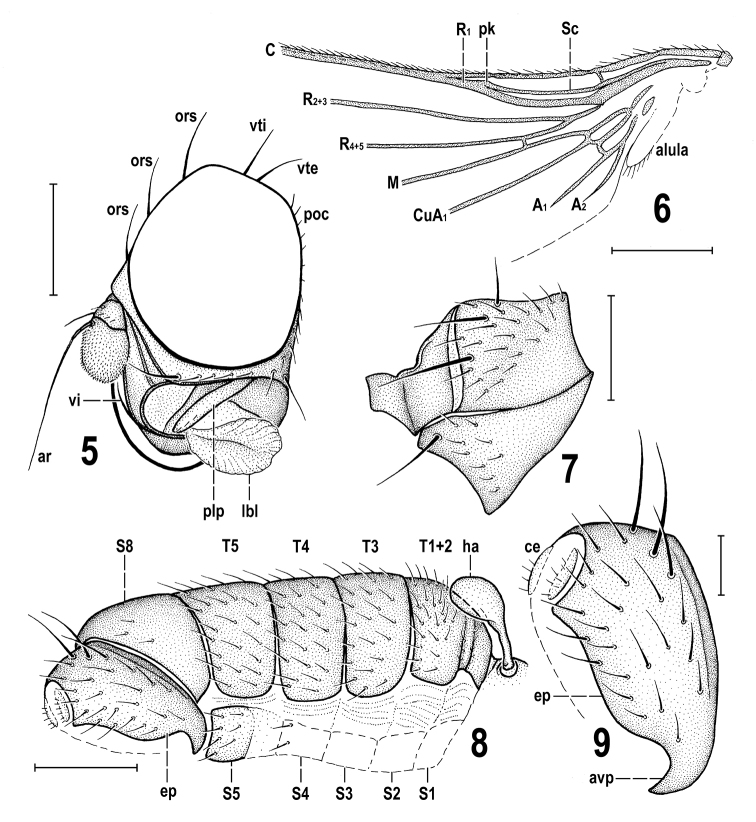
*Protanthomyza grimaldii* sp. nov., holotype male (Baltic amber) **5** head, left sublateral view **6** base of left wing **7** right mesopleuron, sternopleuron and pteropleuron, lateral view **8** abdomen and haltere, right lateral view **9** terminalia, right lateral view. Scale bars: 0.5 mm (**5**); 0.3 mm (**6–8**); 0.1 mm (**9**). For abbreviations see p. 4.

***Head*** (Figs 3–5) higher than long, anteriorly somewhat angularly protruding in front of eye margin (Fig. 5). Occiput very slightly concave, blackish brown. Frons relatively narrow, blackish brown only posteriorly around ocellar triangle, pale brown in anterior half to ochreous yellow at anterior marginal area surrounding frontal lunule. Frontal triangle not developed; ocellar triangle blackish brown, distinctly protruding and delimited by marginal groove. Ocelli large (Figs 3, 4). Orbital plate lighter (ochreous yellow) anteriorly, becoming pale brown posteriorly where contrasting with blackish-brown vicinity of ocellar triangle. Frontal lunule long, ochreous yellow. Face relatively narrow, ochreous, medially lighter and somewhat depressed; parafacialia and anterior half of gena dirty yellow and narrowly brown bordered (gena ventrally); posterior half of gena and postgena brown; mouthparts yellow to ochreous, clypeus pale brown, palpus dirty yellow. Cephalic chaetotaxy (Figs 4, 5): *pvt* (only left one visible) relatively short, convergent but not crossed; *vti* longest of cephalic setae, slightly inclinate; *vte* strongly exclinate and only slightly shorter than *vti*; 3 distinct *ors*, all slightly reclinate, posterior *ors* longest (about as long as *vte*), others becoming slightly shorter anteriorly; *oc* relatively thin (not longer than middle *ors*), proclinate (and unnaturally crossed in holotype), arising inside ocellar triangle; anterior half of frons with about 5 or 6 pairs of microsetae, mostly medially in front of ocellar triangle but a few (1 or 2) also between anterior and middle *ors*; *vi* distinct (Fig. 5), about 3 times as long as foremost peristomal setula; no subvibrissa; 4 or 5 weak proclinate peristomals; postocular setulae in two rows as usual but with only 3 setulae in inner row (Fig. 4); outer row of postoculars long, reaching ventral eye margin; postgena with 2 setae, anterior short, posterior longer. Palpus slender, elongate, with a few (3 visible) minute setulae distally (Fig. 5). Mouthparts relatively short. Eye bare, relatively large and strongly convex, suboval, anteriorly regularly rounded, with only posterior margin somewhat straighter; its longest diameter almost vertical and 1.16 times as long as shortest diameter. Gena low, its shortest height about 0.08 times as long as shortest eye diameter. Antenna medium-sized, generally porrect (Fig. 5) but directed anteroventrally to ventrally, with dark-brown basal segments and pale-brown to ochreous 1^st^ flagellomere. Pedicel with 1 longer seta and several microsetae; 1^st^ flagellomere oval, laterally compressed, with very short, dense and dark pilosity; arista about 1.8 times as long as antenna, entirely bare (Fig. 5), 2 basal segments slightly widened.

***Thorax*** hardly narrower than head, brown to blackish brown, with shining mesonotum and duller pleural part. Mesonotum relatively convex, separated from scutellum by deep suture. Scutellum rounded subtriangular, wider than long, convex dorsally; postscutellum not visible. Thoracic chaetotaxy (see Figs 3, 4, 7) rich as usual in the genus: 1 short *hu* (markedly shorter than anterior *npl*) and only 2 or 3 setulae on humeral callus (= postpronotal lobe); 2 long *npl*, anterior slightly longer; 1 distinct *prs* (about as long as posterior *npl*); 1 long *sa*; 2 *pa*, external very long (longer than *sa*), internal shorter (not visible on left side in Fig. 4); 3 or 4 postsutural *dc* (number different on left and right sides) becoming shorter anteriorly, the hindmost longest (together with apical *sc* longest thoracic setae); *ac* microsetae dense, in 9 or 10 rows on suture, those in medial rows reaching up to posterior *dc*; prescutellar *ac* macrosetae long and strong, as long as *sa*; 2 *sc*, laterobasal shorter (about as long as *prs*), apical very long; no additional setulae on scutellum; 2 upcurved *ppl*, anterior distinct (as long as *hu*), posterior small. Mesopleuron (anepisternum) and sternopleuron (katepisternum) setose (Figs 4, 7) as in most other species of *Protanthomyza*: 3 long *mspl* (1 dorsal upcurved and shortest, 1 posterodorsal and 1 posterior longest, cf. Fig. 7) and numerous setulae in posterodorsal half of mesopleuron; 1 long posterior *stpl* and about 9 or 10 setulae in posterior half of sternopleuron; other sclerites of pleural part of thorax bare.

***Legs*** brown to ochreous, femora darkest, fore coxa pale ochreous. *f_1_* lacking ctenidial spine; 5 (2 longer) distinct widely-spaced setae in posterodorsal row; setae in posteroventral row more numerous but short and weak. *f_2_* with 2 or 3 anterior setae near middle (cf. Fig. 14), otherwise shortly setulose as is *f_3_*. *t_2_* with distinct ventroapical seta (about as long as maximum width of *t_2_*) and 2 or 3 small setae adjacent to the latter; *t_1_*, *t_3_* and all tarsi simply setulose but basitarsi of all legs with ventrobasal setulae somewhat longer than others.

***Wing*** (Figs 1, 6) moderately long and narrow, widest at distal third; veins brown to pale brown, membrane unicolourous, pale-brown tinged; *C* with more or less distinct subcostal break and somewhat attenuated at humeral cross-vein. *C* extended to apex of *M*, densely uniformly setulose on *Cs_2_* (from subcostal break to apex of *R_2+3_*), finely short-pilose more distally (on *Cs_3_* and *Cs_4_*); *Sc* distinct, separate almost along its entire length, only apically fused with *R_1_* to form preapical kink (see Fig. 6, *pk*); *R_1_* short, dilated distally due to fusion with *Sc*; *R_2+3_* long, very slightly sinuate to almost straight and only its extreme apex slightly upcurved to *C*; *R_4+5_* slightly recurved in distal half, divergent from *R_2+3_* and apically slightly convergent with *M*; *M* almost straight. Cell *dm* of moderate length, narrow proximally and much widened distally, with angle of anterior outer corner obtuse while that of posterior outer corner distinctly acute (cf. Fig. 12); *r-m* situated in basal third of cell *dm*; *dm-cu* straight; apical portion of *CuA_1_* much shorter than distance between *r-m* and *dm-cu*, distinctly longer than *dm-cu* and almost reaching wing margin; *A_1_* relatively long but ending far from wing margin; *A_2_* well developed, slightly shorter than *A_1_* (Fig. 6); alula distinct but narrow (Fig. 6). Wing measurements: length ca 2.4 mm, width ca 0.8 mm, *Cs_3_* : *Cs_4_* = 1.38, *r-m\dm-cu* : *dm-cu* = 3.50, *r-m\dm-cu* : *CuA_1_* = 2.50. Haltere (Figs 4, 8) pale ochreous, knob relatively large, darker dorsally.

***Abdomen*** (Figs 1, 2, 8) relatively short, robust but not very broad. Preabdominal terga blackish brown; *T1* fused with *T2* but delimited by a groove (Fig. 8); *T1* finely short-setose, *T2–T5* with relatively long and dense setae (Fig. 8); *T2* somewhat shorter than *T3*, *T3–T5* subequal in length, all relatively short and transverse (Figs 2, 8). Preabdominal sterna mostly invisible (Figs 2, 8) but probably small (narrow) and pale-pigmented; only *S5* discernible (Fig. 8), paler brown and shorter than adjacent *T5*, with sparse setae. Also 2 setae of *S4* visible on right side of abdomen (Fig. 8) but margins of sclerite are not recognizable. Postabdomen: *S6* and *S7* not visible because situated on obscured left side of abdomen (cf. Fig. 1), probably asymmetrical and (partly) fused together (dorsally also with *S8*) as in other *Protanthomyza* species (cf. Roháček 2013a, fig. 4H). *S8* relatively long, blackish brown, situated dorsally and readily visible on right side (Figs 2, 8), almost bare, with only a few setulae.

***Genitalia*.** Epandrium (Figs 8, 9) relatively long but tapered posteriorly, with anteroventral corner modified to distinct, flat and somewhat hook-like projection (see Fig. 9, *avp*) distinctly different from those in other *Protanthomyza* species where known (cf. Roháček 2013a, figs 1F, 2E, 4H, 6B, 12C). Epandrium anterodorsally with 3 pairs of long erect setae (the most lateral markedly shorter), otherwise with scattered short setae. Anal fissure relatively small and cercus reduced (small and narrow) with fine short pubescence (Fig. 9). Gonostylus not discernible (on any side) but probably small and pale. No structures of internal genitalia visible.

**Figures 10, 11.**
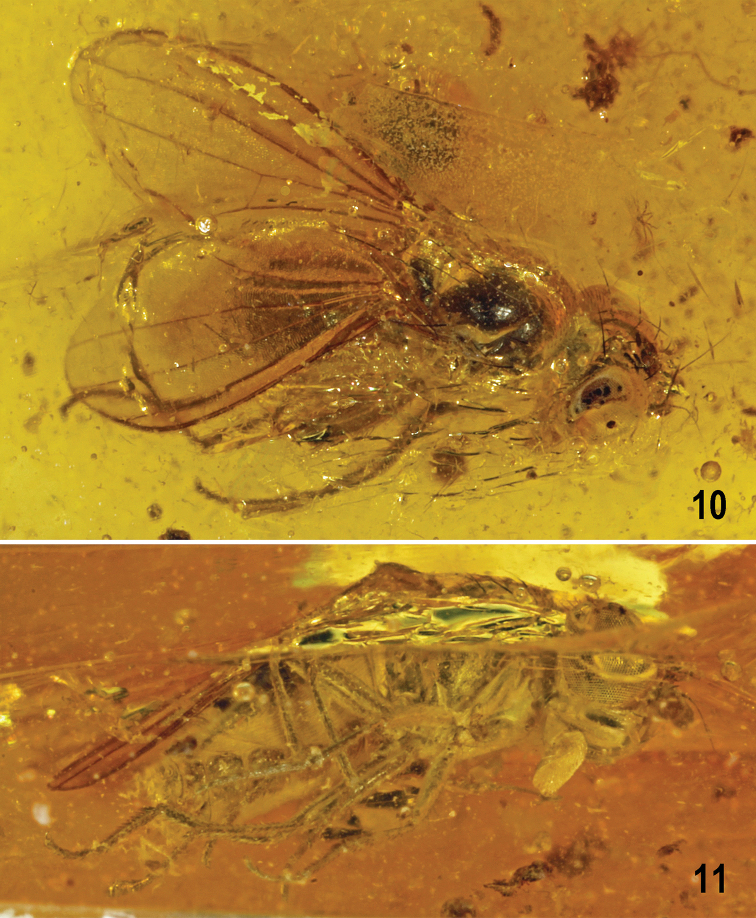
*Protanthomyza grimaldii* sp. nov., female paratype (Baltic amber) **10** entire specimen, subdorsal view **11** ditto, lateroventral view. Body length ca 2.9 mm. Photographs by J. Roháček (adapted from Roháček 2013a, fig. 7E, F).

**Figures 12–14.**
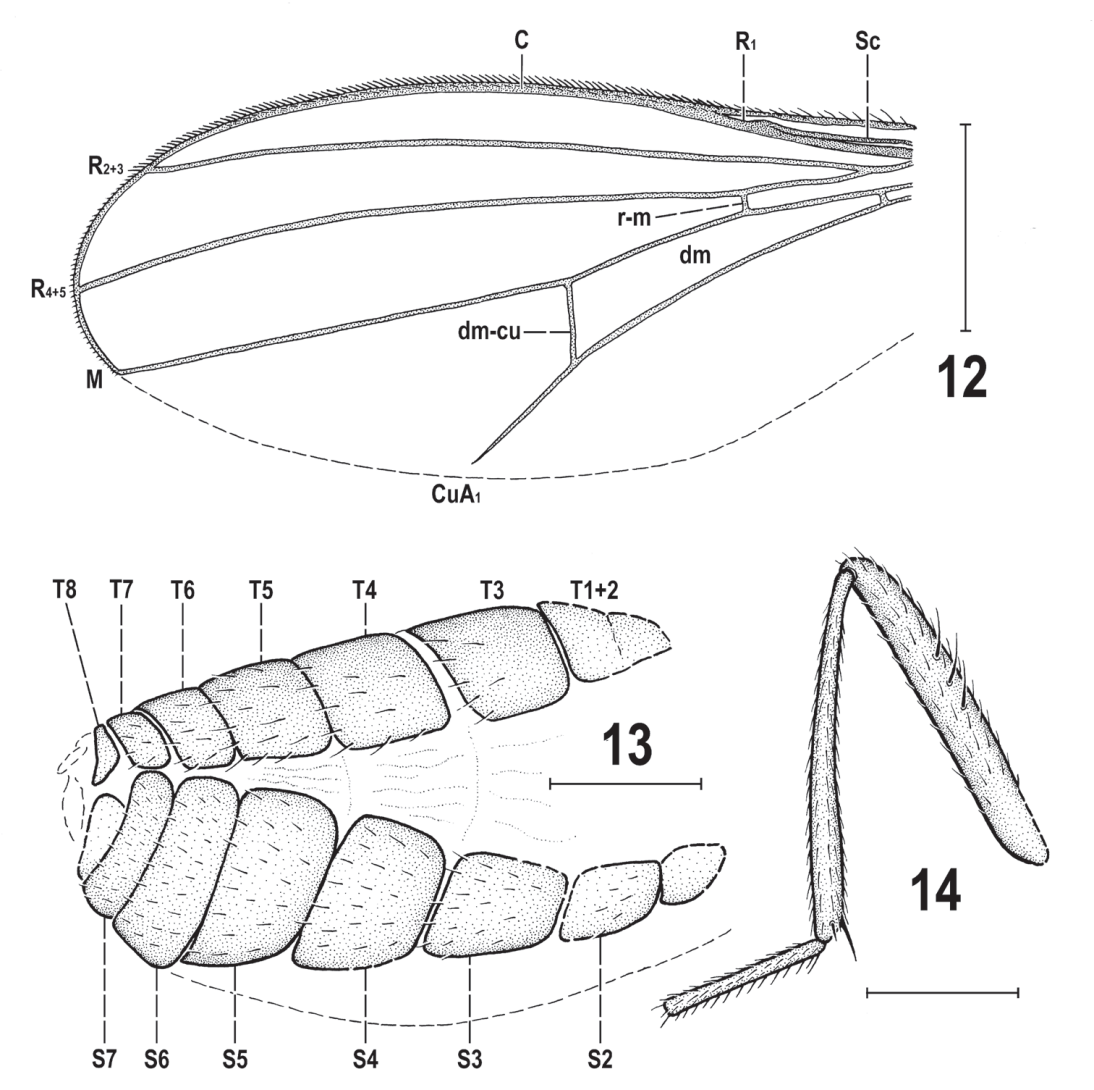
*Protanthomyza grimaldii* sp. nov., female paratype (Baltic amber) **12** left wing (invisible parts omitted) **13** abdomen, right lateroventral view **14** right f_2_, t_2_ and mid basitarsus, anterior view. Scale bars: 0.5 mm (**12**), 0.3 mm (**13**, **14**). For abbreviations see p. 4.

**Female** (Figs 10, 11). Similar to male unless mentioned otherwise. Total body length ca 2.9 mm. *pvt* short, strongly convergent, with apices meeting medially; *oc* proclinate and divergent; setae in posteroventral corner of postgena subequal. Palpus with more setulae (5 or 6) visible ventrally, subapically and apically. Mesonotum with 3 strong *dc* in left row and with 4 *dc* (2 posterior strong, 2 anterior short and weaker) in right row; prescutellar *ac* macrosetae longer than *sa*; about 8 rows of *ac* microsetae on suture. Legs more slender but with same chaetotaxy as in male, except t_2_ (Fig. 14) with ventroapical seta longer. Wing (Fig. 12) venation resembling that of male but cell *dm* shorter and cross-vein r-m situated more distally. Wing measurements: length 2.58 mm, width 0.95 mm, *Cs_3_* : *Cs_4_* = 1.44, *rm\dm-cu* : *dm-cu* = 2.37, *rm\dm-cu* : *CuA_1_* = 1.97.

***Abdomen*** (Figs 11, 13) only partly visible. Preabdomen with terga (*T1+2, T3–T5*) obviously darker brown than sterna, relatively narrow, hence pleural part of preabdominal segments large; setae on *T3–T5* (and also on *T6* and *T7*) longer than those on adjacent sterna. Preabdominal sterna pale brown to ochreous. *S1–S5* becoming distinctly wider posteriorly, *S5* widest and largest.

***Postabdomen*.***T6* distinctly shorter than *T5*, transverse; *T7* yet shorter and also narrower than *T6*. *S6* strikingly broad and transverse, wider but shorter than *S5* and apparently larger than adjacent *T6* and laterally almost reaching the latter; *S7* only half length of *S6*, strongly transverse and laterally meeting with sides of *T7* (Fig. 13); both *S6* and *S7* finely setulose. Apex of postabdomen obscured, only *T8* discernible as small bare(?) sclerite (Fig. 13), less than half length of *T7*. Cercus not visible.

## Discussion

*Protanthomyza grimaldii* sp. nov. was previously recognized as a new species by Roháček (2013a: 451) but left unnamed because of insufficient material (a single female having a number of characters not visible). Thanks to the efforts of Christel Hoffeins, a male specimen conspecific with this female was recently obtained for examination and enabled the description and naming of this species. This is the third species of *Protanthomyza* where both sexes are known; formerly, the male and female were described only in *P. krylovi* (1 male and 2 females found in one piece of Baltic amber) and *P. tschirnhausi* (1 male and 1 female in separate pieces of Bitterfeld amber), see Roháček (2013a).

*Protanthomyza grimaldii* belongs to a group of species lacking a ctenidial spine on the fore femur and both sexes are correctly keyed by Roháček (2013a: 442, as *P.* sp. nov.). With its bare arista and generally similar chaetotaxies of the head and thorax, it most closely resembles *P. loewi* (known only from the female), which could be its nearest relative. However, *P. grimaldii* can be easily distinguished from *P. loewi* by the distinctly smaller 1^st^ flagellomere of the antenna (Fig. 5, cf. Roháček 2013a, fig. 8B), the ocellar triangle delimited by a groove (Fig. 4), the less elongate wing with more divergent *R_2+3_* and *R_4+5_* (Fig. 12, cf. Roháček 2013a, fig. 7D) and the shorter and wider female abdomen with short T7 and broadly transverse S6 and S7 (Fig. 13, cf. Roháček 2013a, fig. 8A), apart from other smaller dissimilarities in head colouration, length of *ppl* setae, *f_1_* and *t_2_* chaetotaxy, etc. In the male, *P. grimaldii* differs from all five other species where the male is known (cf. Roháček 2013a) by the elongate and posteriorly tapered epandrium with a robust, hook-like, curved anteroventral process (Fig. 9, *avp*). Only in *P. tschirnhausi* is the epandrial process similarly robust and flat but it is simply triangular (not hooked) and the epandrium is short, almost globose (not elongate) (cf. Roháček 2013a, fig. 12C). Moreover, *P. tschirnhausi* differs markedly from *P. grimaldii* in a number of other characters including the ciliate arista, very large eyes, only 2 *dc* and a strong ctenidial spine on *f_1_*, see Roháček (2013a). Unfortunately, the male of *P. loewi* remains unknown and, consequently, the previously suggested relationship of that species with *P. grimaldii* cannot be confirmed by examination of characters of the male terminalia.

## Conclusions

Based on the data presented above, it can be concluded that:

(1) *P. grimaldii* is the ninth named species of the genus *Protanthomyza*, making this genus the most species-rich fossil genus of Anthomyzidae.

(2) The description of a new *Protanthomyza* species extends the morphological diversity evident in the male and female terminalia of this genus. This adds to the previously documented wide morphological diversity among species seen in the head structures, chaetotaxies, and formation of the sclerites of the abdomen besides the male and female terminalia.

(3) Two anal veins (*A_1_*, *A_2_*) can continue to be treated as a subfamily/generic character for Protanthomyzinae and *Protanthomyza*, respectively.

(4) The chaetotaxy of the mesopleuron (usually with 3 posterior *mspl* macrosetae + numerous setulae more anteriorly) and sternopleuron (with 1 posterior *stpl* macroseta + setulae) seems to be rather uniform within the genus, although in a few species some setulae on the mesopleuron can be enlarged and/or one (usually the dorsal) *mspl* macroseta is reduced (cf. Roháček 1913a).

(5) The anteroventral process of the epandrium is a synapomorphic character of *Protanthomyza*. It is expected that this process will be found in all other species where the male is unknown, viz. in *P. loewi*, *P. meunieri* and *P. presli*.

## Supplementary Material

XML Treatment for Protanthomyza

XML Treatment for Protanthomyza grimaldii
